# Disrupting reconsolidation by PKA inhibitor in BLA reduces heroin-seeking behavior

**DOI:** 10.3389/fncel.2022.996379

**Published:** 2022-08-29

**Authors:** Yanghui Zhang, Haoxian Li, Ting Hu, Zijin Zhao, Qing Liu, Haoyu Li

**Affiliations:** ^1^Center of Medical Genetics, Jiangmen Maternity and Child Health Care Hospital, Jiangmen, China; ^2^Department of Neurosurgery, Xiangya Hospital, Central South University, Changsha, China; ^3^The Institute of Skull Base Surgery and Neurooncology at Hunan Province, Changsha, China

**Keywords:** reconsolidation, heroin, PKA, BLA, drug-seeking

## Abstract

Drug abuse is considered a maladaptive pathology of emotional memory and is associated with craving and relapse induced by drug-associated stimuli or drugs. Reconsolidation is an independent memory process with a strict time window followed by the reactivation of drug-associated stimulus depending on the basolateral amygdala (BLA). Pharmacology or behavior treatment that disrupts the reconsolidation can effectively attenuate drug-seeking in addicts. Here, we hypothesized that heroin-memory reconsolidation requires cAMP-dependent protein kinase A (PKA) of BLA based on the fundamental effect of PKA in synaptic plasticity and memory process. After 10 days of acquisition, the rats underwent 11 days of extinction training and then received the intra-BLA infusions of the PKA inhibitor Rp-cAMPS at different time windows with/without a reactivation session. The results show that PKA inhibitor treatment in the reconsolidation time window disrupts the reconsolidation and consequently reduces cue-induced reinstatement, heroin-induced reinstatement, and spontaneous recovery of heroin-seeking behavior in the rats. In contrast, there was no effect on cue-induced reinstatement in the intra-BLA infusion of PKA inhibitor 6 h after reactivation or without reactivation. These data suggest that PKA inhibition disrupts the reconsolidation of heroin-associated memory, reduces subsequent drug seeking, and prevents relapse, which is retrieval-dependent, time-limited, and BLA-dependent.

## Introduction

Exposure to drug-associated stimuli precipitates the craving and relapse, which presents a major challenge to drug abstinence (Childress et al., 1999; Crombag et al., 2008; Chen et al., 2021b). The drug-associated stimuli and reward-associated memories contributed to the craving, compulsive opioid taking, and seeking in addicts (O'Brien et al., 1992; Kilts et al., 2001; Milton and Everitt, 2012; Vandaele and Janak, 2018). The memory of these stimuli, powerfully maintaining conditioned reinforcing properties *via* the associations between the value of the drug and aversive effects, is highly difficult to eliminate in drug addicts and laboratory animals (de Wit and Stewart, 1981; Gawin, 1991; Di Ciano and Everitt, 2004; Milton and Everitt, 2012; LeCocq et al., 2020; Baidoo and Leri, 2022). Thus, disrupting drug memory through various interventions is a potential strategy to prevent relapse (Milton and Everitt, 2010; Correia et al., 2020; Barak and Goltseker, 2021).

Pioneering research over the past two decades demonstrates that targeted interference with reconsolidation—a *de novo* protein synthesis-dependent and time-dependent memory process occurred when memory is reactivated—could attenuate the heroin-associated memory and reduce relapse (Nader et al., 2000a; Dudai, 2006; Tronson et al., 2006), indicating that it is critical to explore the specific mechanisms of heroin-memory reconsolidation with a focus on promising therapeutic targets. Numbers of studies have developed several potential pharmacological interventions for the treatment of maladaptive memory that are consistent with the characteristic of reconsolidation (Sara, 2000; Debiec and Ledoux, 2004; Schiller et al., 2010; Xue et al., 2017; Chen et al., 2021a; Zhang et al., 2021; Xie et al., 2022). However, fewer of them translated into clinics, so the development of an effective therapeutic strategy is urgent.

One of the well-studied kinases in eukaryotic biology is cAMP-protein kinase A (PKA), which is important for synaptic plasticity and is involved in many memory processes, especially some forms of reconsolidation in BLA (Huang and Kandel, 1998; Rammes et al., 2000). Pharmacological studies have confirmed the role of PKA in the formation of long-term memory (Bernabeu et al., 1997; Bourtchouladze et al., 1998; Ahi et al., 2004; Quevedo et al., 2004). PKA has also been demonstrated to play a role in synaptic tagging and counterpart behavior of synaptic tagging (Young et al., 2006; Moncada et al., 2011; Rabinovich Orlandi et al., 2020). Importantly, the role of PKA in reconsolidation has been demonstrated by Tronson et al. in auditory fear memories (Tronson et al., 2006). In addition, some studies verified that PKA affects other memory forms of reconsolidation, such as contextual drug memory and conditioned taste aversion memory (Koh and Bernstein, 2003; Sanchez et al., 2010). The activity of PKA regulates Pavlovian associative memories, such as methamphetamine-, amphetamine-, cocaine-, and morphine-induced reward memories in the conditioned place preference (CPP) model and response-outcome associative learning in the cocaine self-administration (SA) model (Sharifzadeh et al., 2006; Gerdjikov et al., 2007; Sanchez et al., 2010; Arguello et al., 2014; Yang et al., 2020). Nevertheless, several critical gaps remain in our understanding of PKA's role in the reconsolidation of memory. First, it is unknown whether the PKA activity regulates the reconsolidation of heroin-associated memory. Second, it is unclear that the PKA has a similar effect on the reconsolidation of Pavlovian associative memories vs. response-outcome instrumental memories since the available literature shows that Pavlovian associative and response-outcome instrumental memories are reconsolidated *via* partially different neural mechanisms (Miller and Marshall, 2005; Wells et al., 2016; Bender and Torregrossa, 2020).

The amygdala, especially the basolateral amygdala (BLA), plays a critical role in the reconsolidation of reward memory as well as heroin-associative learning, which is associated between appetitive events and a neutral stimulus (See et al., 2003; Hellemans et al., 2006; Yuan et al., 2020). Hence, treatment targeting heroin-associated memory reconsolidation of specific targets in BLA seems a viable strategy to maintain long-term abstinence and prevent relapse.

In this study, we tested the hypothesis that whether PKA in the BLA engages in the reconsolidation of heroin-associated memory using a self-administration model. Furthermore, we tested the role of PKA inhibition during reconsolidation on subsequently cue-induced, heroin-induced reinstatement, and spontaneous recovery of heroin-seeking behavior.

## Materials and methods

### Animals

Male Sprague Dawley rats were housed in a group of 4–5 per cage with constant temperature (24 ± 2°C) and humidity (55 ± 5%) and free access to food and water in a reversed 12-h light/dark cycle (lights on at 7:30 p.m.). To reduce the stress response of the rats, all experimental animals were handled for 5 min by the experimenter per day for 5 days before the surgery. All animal experiments were approved by the Xiangya Hospital Ethics Committee, Xiangya Hospital (Changsha, China), and carried out during the dark phase (7:30 a.m.−7:30 p.m.).

### Surgery

The rats were anesthetized using sodium pentobarbital (60 mg/kg, i.p.). Jugular catheters were implanted into the right jugular vein and bilateral guide cannulae targeting the BLA (AP: −2.8 mm, ML: ± 5.0 mm, DV: −8.5 mm relative to bregma) as described previously (Wu et al., 2011; Xie et al., 2022). The cannulae were fixed with four stainless steel screws and impregnated with dental cement in the skull. After surgery, the rats were housed individually to recover for 5–7 days before the start of experimentation.

### Behavioral procedures

#### Self-administration training

The training paradigm was the same as in our previous studies (Xie et al., 2022). The rats were trained in the operant chambers (AniLab Software & Instruments, Ningbo, China). The chamber was equipped with two nosepoke operandi (active/inactive and located adjacent to the right wall). Rats underwent self-administer heroin (0.05 mg/kg/infusion) training in three 1-h sessions for 10 days under a fixed-ratio 1 (FR1) schedule of reinforcement on the active nosepoke. Nosepokes in active operandi led to heroin infusions accompanied by a 5-s tone-light cue, and we limited the maximum number of heroin infusions to 20/h to prevent overdose (Xue et al., 2012; Luo et al., 2015). The rats had a 10 s timeout period between infusions. On the contrary, no result in any stimulus or infusions when inactive nosepoke was contacted. At the beginning of each session, a house light will illuminate during the entire session to remain on. Moreover, seven rats were excluded from all experiments due to catheter occlusion or failure to acquire self-administer with heroin.

#### Extinction

The 3-h daily extinction sessions were performed for nine continuous days after the self-administration training. The extinction session was the same as the self-administration training except for contact operandi without programmed consequences (cue stimulus or infusions), and the house light above the nosepoke operandi was not illuminated for the entire session. A 3-h daily cue extinction was performed in experiments 1, 3, and 4, the conditions were the same as during heroin self-administration training, except that there was no heroin intravenous delivery.

#### Reactivation

A 15-min reactivation was performed 24 h after the last extinction day in experiments 1, 2, and 4. The conditions of the reactivation session were the same as acquisition training except that after contact, active nosepoke only cues were delivered but not heroin.

#### Cue-induced reinstatement test

The rats returned to the original chamber for 1 h and recorded the numbers of the active nosepoke, during which the conditions were the same as the reactivation session.

#### Spontaneous recovery test

After the last extinction session, the rats were housed for 28 days and returned to the original chamber for 1 h for a spontaneous recovery test. The conditions were the same as the cue-induced reinstatement test.

#### Drug-priming reinstatement test

The rats were injected with a low dose of heroin (0.25 mg/kg, s.c.) 5 min before the 1-h drug-priming reinstatement test. The conditions of the test were the same as in the reactivation session.

### Statistical analysis

The experimental data were analyzed by GraphPad, v.8.0. All data were analyzed by two-way ANOVAs with a between-subjects factor of treatment condition and a within-subjects factor of test condition followed by the Bonferroni *post-hoc* analysis. The data were presented as mean ± SEM, and a *p* < 0.05 was considered statistically significant.

## Experiment design

### Experiment 1: The effect of immediately post-reactivation intra-BLA PKA inhibition on the subsequent cue- and heroin-induced reinstatement tests

**Figure 1:**
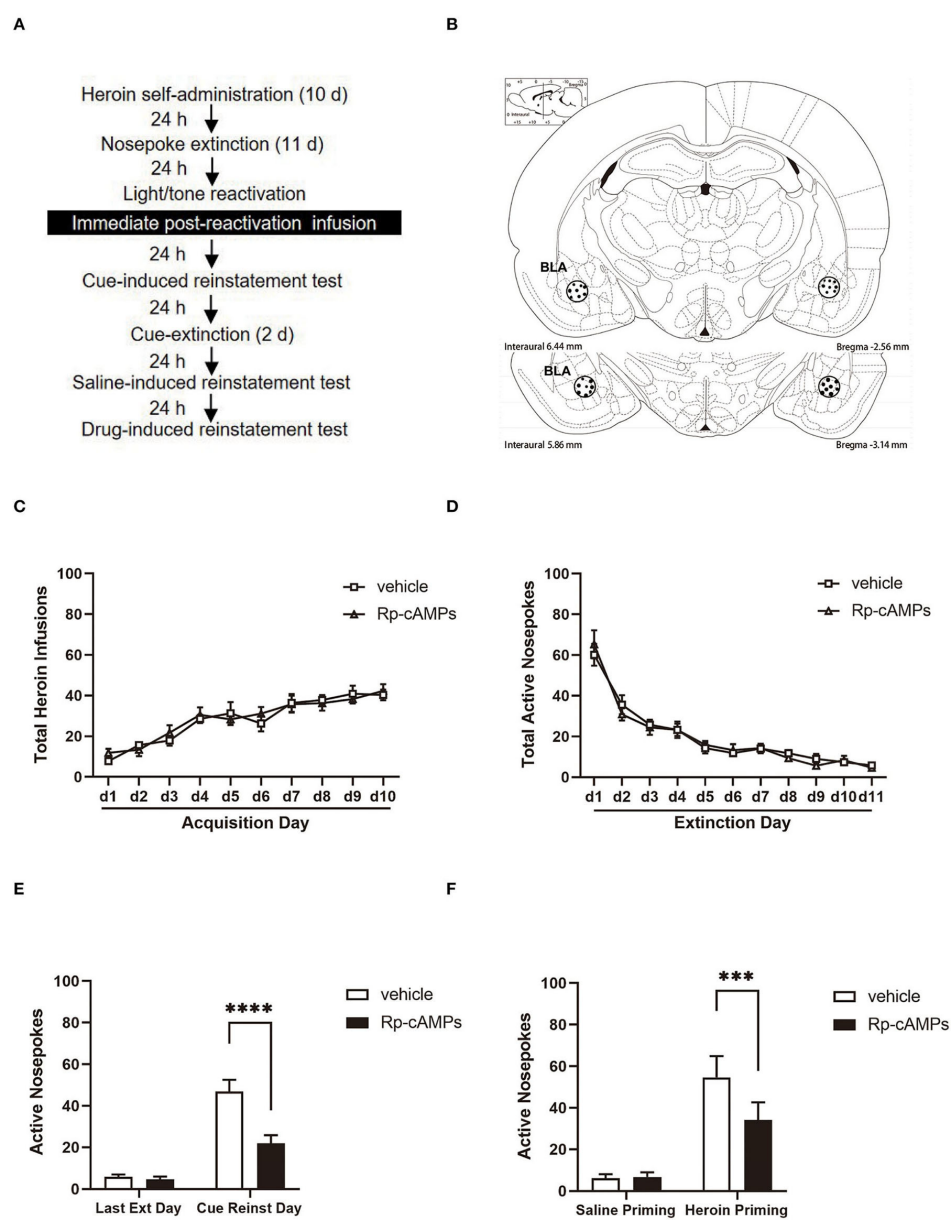
The effect of immediately post-reactivation intra-basolateral amygdala (BLA) protein kinase A (PKA) inhibition on the subsequent cue- and heroin-induced reinstatement tests. **(A)** Timeline of the experimental procedure. **(B)** The regions of the basolateral amygdala (BLA: −2.8 mm from bregma) into which the cannula was placed as shown in the rostral faces of each coronal section. **(C)** The total number of infusions during the acquisition of heroin self-administration. **(D)** The total number of active nosepoke responses during extinction sessions. **(E)** Active nosepoke responses during the last day of extinction and the cue-induced reinstatement test. **(F)** Active nosepoke responses during the saline- or heroin- primed reinstatement test. The value of *n* = 8 rats per group. Data are means ± SEM, ***p < 0.001, ****p < 0.0001, compared with the vehicle group. Ext, extinction; Reinst, reinstatement.

In experiment 1, we first investigated the effect of **an** immediate intra-BLA infusion of Rp-cAMPS after reactivation on subsequent cue- and heroin-induced reinstatement of heroin-seeking behavior. The infusion dose of Rp-cAMPS was based on previous studies (Tronson et al., 2006; Sanchez et al., 2010). The rats underwent heroin self-administration for 10 days, followed by extinction training for 11 consecutive days in the same original box. A 15-min reactivation session was performed 24 h after the last extinction session. Then, the rats received bilateral intra-BLA infusion with Rp-cAMPS or vehicle (18 μg per side) immediately after the stimulus reactivation. Then, 24-h after the infusion, the rats were tested for cue-induced reinstatement and then trained in cue extinction sessions for 2 days. After 24 h, the rats were tested for heroin-induced reinstatement (Figure 1A).

### Experiment 2: The effect of immediate post-reactivation intra-BLA PKA inhibition on the cue-induced reinstatement and spontaneous recovery tests 28 days later

**Figure 2:**
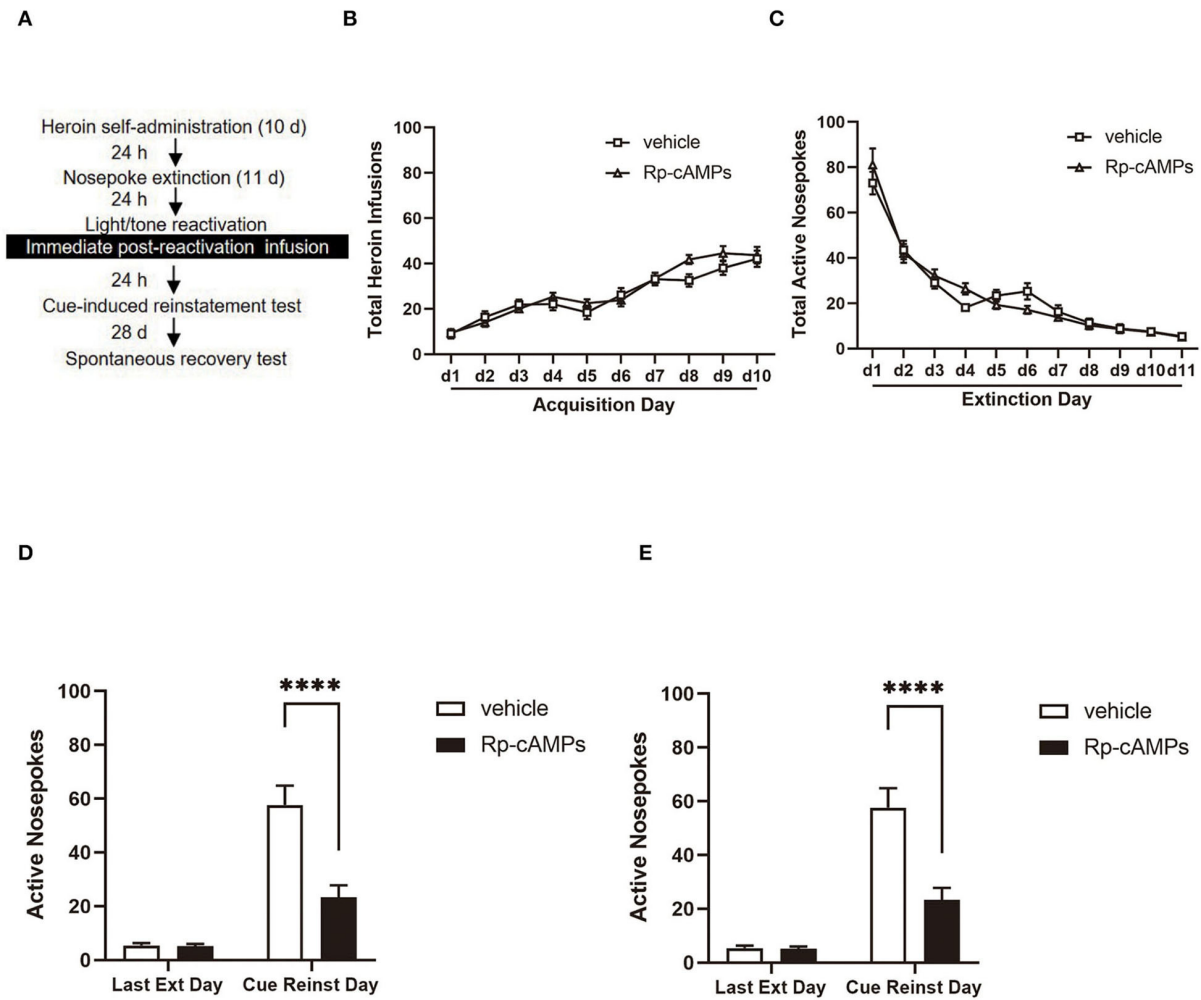
The effect of immediately post-reactivation intra-BLA PKA inhibition on cue-induced reinstatement and spontaneous recovery test 28 days later. **(A)** Timeline of the experimental procedure. **(B)** The total number of infusions during the acquisition of heroin self-administration. **(C)** The total number of active nosepoke responses during extinction sessions. **(D)** Active nosepoke responses during the last day of extinction and the cue-induced reinstatement test. **(E)** Active nosepoke responses during the last extinction day and the spontaneous recovery test. The value of *n* = 9 rats per group. Data are means ± SEM, ****p < 0.0001, compared with the vehicle group. Ext, extinction; Reinst, reinstatement; SR, spontaneous recovery.

In experiment 2, we test the effect of immediate intra-BLA infusion of Rp-cAMPS after reactivation on the subsequent cue-induced reinstatement and spontaneous recovery of heroin-seeking behavior. The rats underwent heroin self-administration for 10 days and followed by extinction training for 11 consecutive days in the same original box. A 15-min reactivation session was performed 24 h after the last extinction session. Next, the rats received bilateral intra-BLA infusion with Rp-cAMPS or vehicle (18 μg per side) immediately after the stimulus reactivation. Then, the rats were tested in cue-induced reinstatement 24 h after the infusion. Twenty-eight days after the test, the rats were tested for spontaneous recovery (Figure 2A).

### Experiment 3: The effect of intra-BLA PKA inhibition without reactivation on the subsequent cue- and heroin-induced reinstatement tests

**Figure 3:**
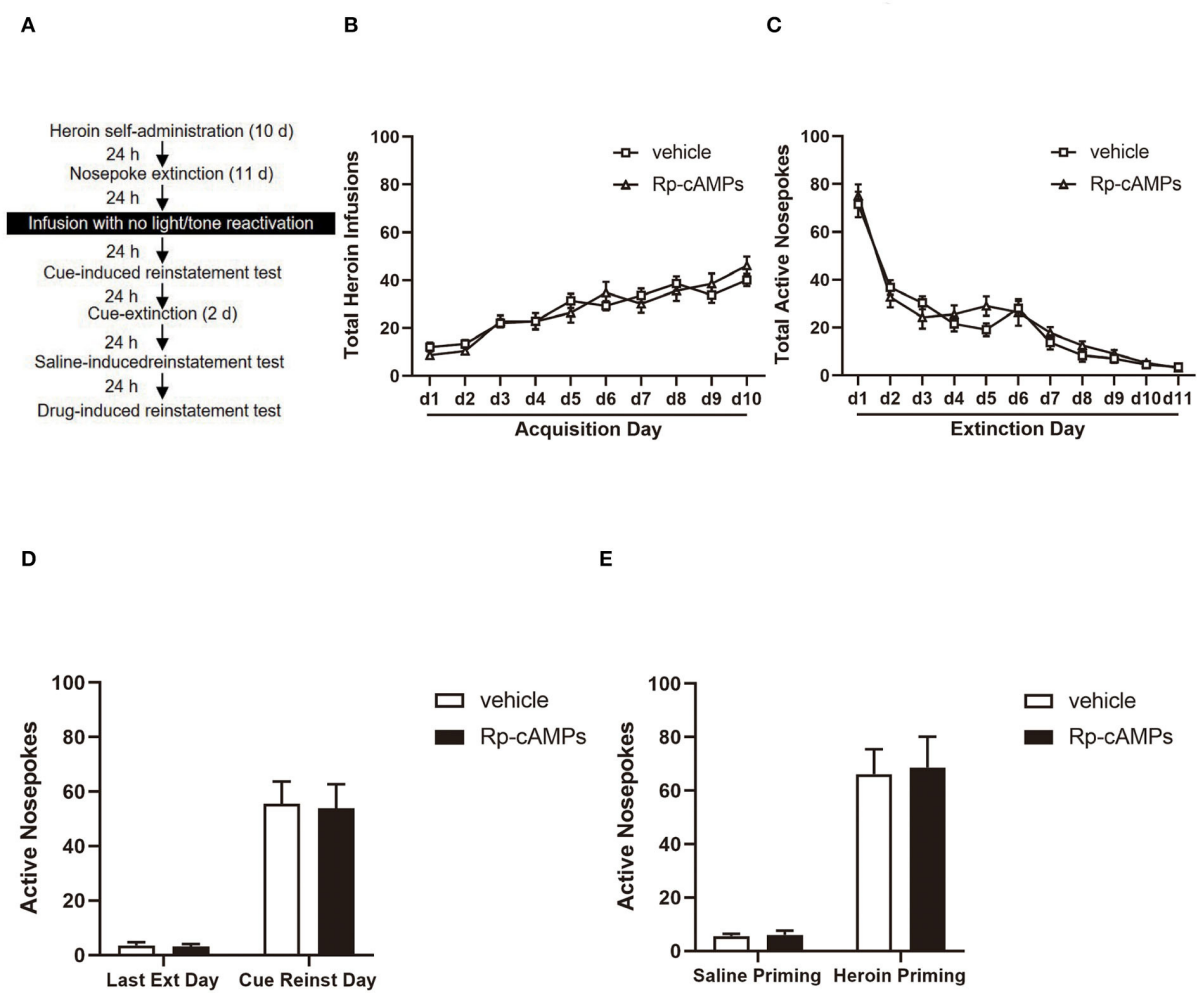
The effect of intra-BLA PKA inhibition without reactivation on the subsequent cue- and heroin-induced reinstatement tests. **(A)** Timeline of the experimental procedure. **(B)** The total number of infusions during the acquisition of heroin self-administration. **(C)** The total number of active nosepoke responses during extinction sessions. **(D)** Active nosepoke responses during the last day of extinction and the cue-induced reinstatement test. **(E)** Active nosepoke responses during the saline- or heroin-primed reinstatement tests. The value of *n* = 8 rats per group. Data are means ± SEM. Ext, extinction; Reinst, reinstatement.

The procedure of experiment 3 was the same as in experiment 1, except that the rats received a bilateral intra-BLA infusion of Rp-cAMPS with a no-reactivation session (Figure 3A).

### Experiment 4: The effect of delayed reactivation of intra-BLA PKA inhibition on the subsequent cue- and heroin-induced reinstatement tests

**Figure 4:**
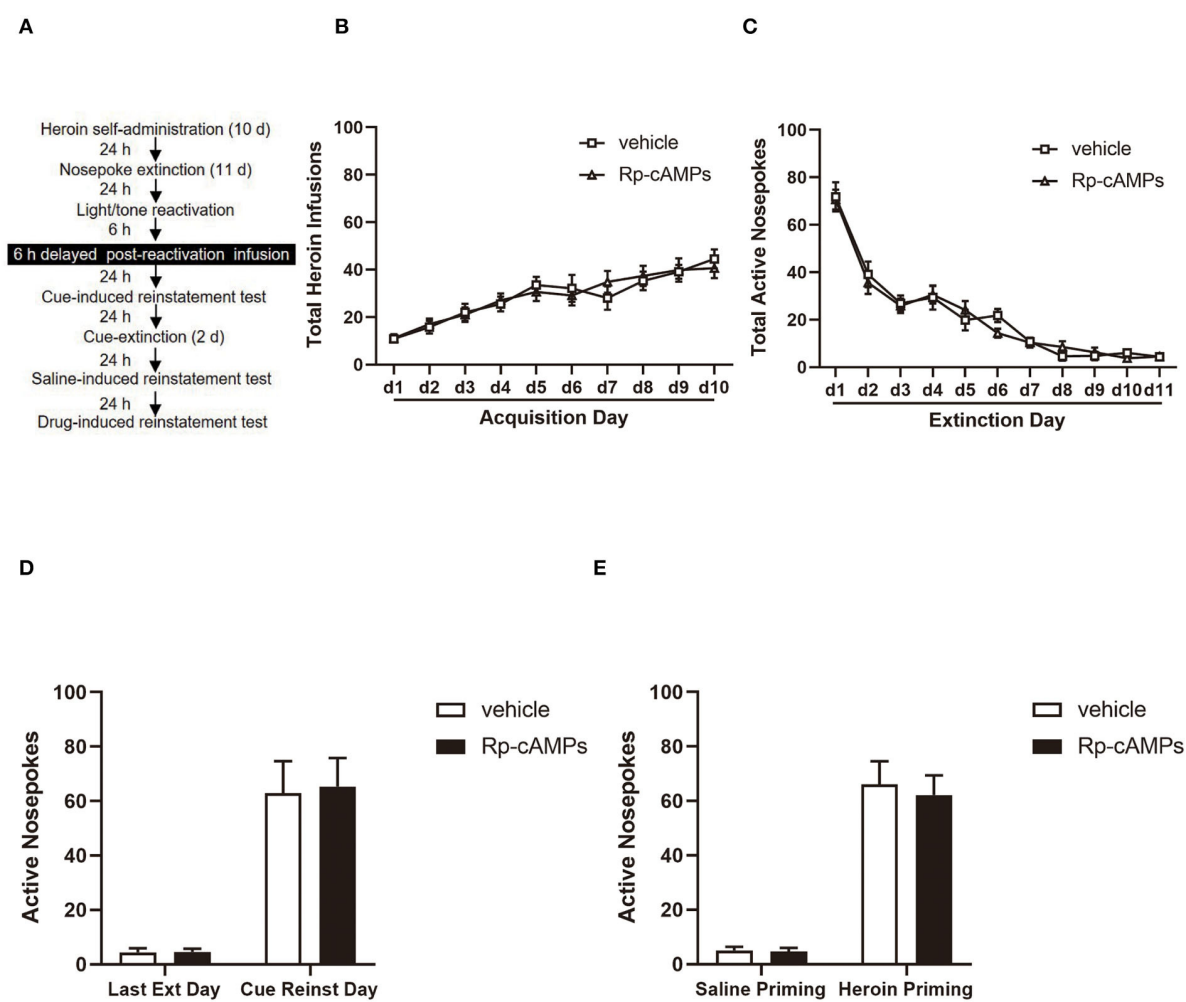
The effect of delayed reactivation of intra-BLA PKA inhibition on the subsequent cue- and heroin-induced reinstatement tests. **(A)** Timeline of the experimental procedure. **(B)** The total number of infusions during acquisition of heroin self-administration. **(C)** The total number of active nosepoke responses during extinction sessions. **(D)** Active nosepoke responses during the last day of extinction and the cue-induced reinstatement test. **(E)** Active nosepoke responses during the saline- or heroin-primed reinstatement tests. The value of *n* = 8 rats per group. Data are means ± SEM. Ext, extinction; Reinst, reinstatement.

The procedure of experiment 4 was the same as in experiment 1, except that the rats received a bilateral intra-BLA infusion of Rp-cAMPS 6 h after the reactivation session (Figure 4A).

## Results

### Intra-BLA PKA inhibition following reactivation reduces subsequent cue- and drug-induced reinstatement

All cannulae were implanted 1 mm above the BLA (Figure 1B). Infusion numbers of acquisition sessions of heroin self-administration were not different between the groups that followed infusion with Rp-cAMPS (*N* = 8) or vehicle (*N* = 8) after reactivation [main effect of the trial session: *F*_(9, 126)_ = 29.38, *p* < 0.001; main effect of the treatment condition: *F*_(1, 14)_ = 0.066, *p* = 0.801; interaction of trial session × treastment condition: *F*_(9, 126)_ = 0.585, *p* = 0.807; Figure 1C]. In addition, there are no differences between the groups across the 11 days of extinction session [main effect of the trial session: *F*_(10, 140)_ = 67.56, *p* < 0.001; main effect of the treatment condition: *F*_(1, 14)_ = 0.027, *p* = 0.872; interaction of trial session × treatment condition: *F*_(10, 140)_ = 0.447, *p* = 0.921; Figure 1D]. There was a significant difference in the interaction between active nosepoke in the Rp-cAMPS and vehicle groups in the subsequent cue-induced reinstatement test (*p* < 0.001; Figure 1E). Importantly, the Rp-cAMPS group following reactivation also resulted in a subsequent decrease in drug-induced reinstatement compared with the vehicle group (*p* < 0.001; Figure 1F). Thus, immediate intra-BLA infusion of PKA inhibition after reactivation is seen to disrupt reconsolidation and reduce subsequent heroin-seeking behavior in the rats.

### Intra-BLA PKA inhibition following reactivation decreases spontaneous recovery of heroin-seeking behavior

All cannulae were implanted 1 mm above the BLA. Infusion numbers of acquisition sessions of heroin self-administration were not different between the groups that followed infusion with Rp-cAMPS (*N* = 9) or vehicle (*N* = 9) after reactivation [main effect of the trial session: *F*_(9, 144)_ = 53.76, *p* < 0.001; main effect of the treatment condition: *F*_(1, 16)_ = 0.704, *p* = 0.414; interaction of trial session × treatment condition: *F*_(9, 144)_ = 1.595, *p* = 0.122; Figure 2B]. There are no differences between the groups across the 11 days of extinction session [main effect of the trial session: *F*_(10, 140)_ = 130.5, *p* < 0.001; main effect of the treatment condition: *F*_(1, 14)_ = 0.003, *p* = 0.958; interaction of trial session × treatment condition: *F*_(10, 140)_ = 1.778, *p* = 0.07; Figure 2C]. There was a significant difference in the interaction between active nosepoke in the Rp-cAMPS and vehicle groups in the subsequent cue-induced reinstatement test (*p* < 0.001; Figure 2D). The Rp-cAMPS group following reactivation also resulted in a subsequent decrease in the spontaneous recovery test of heroin-seeking behavior, compared with the vehicle group (*p* < 0.001; Figure 2E). Therefore, immediate intra-BLA infusion of PKA inhibition after reactivation has long-lasting effects on reducing heroin-seeking behavior in the rats.

### Intra-BLA PKA inhibition without reactivation has no effect on subsequent cue- and drug-induced reinstatement

All cannulae were implanted 1 mm above the BLA. Infusion numbers of acquisition sessions of heroin self-administration were not different between the groups that followed infusion with Rp-cAMPS (*N* = 8) or vehicle (*N* = 8) [main effect of the trial session: *F*_(9, 126)_ = 33.26, *p* < 0.001; main effect of the treatment condition: *F*_(1, 14)_ = 0.001, *p* = 0.971; interaction of trial session × treatment condition: *F*_(9, 126)_ = 1.247, *p* = 0.273; Figure 3B]. There are also no differences between the groups across the 11 days of extinction session [main effect of the trial session: *F*_(10,140)_ = 92.3, *p* < 0.001; main effect of the treatment condition: *F*_(1, 14)_ = 0.361, *p* = 0.558; interaction of trial session × treatment condition: *F*_(10, 140)_ = 1.175, *p* = 0.312; Figure 3C]. There was no significant difference in the interaction between active nosepoke in the Rp-cAMPS and vehicle groups in the subsequent cue- (*p* > 0.1; Figure 3D) and drug-induced reinstatement test (*p* > 0.1; Figure 3E). In summary, the results indicated that the effect of PKA inhibition depends upon the reactivation session.

### Intra-BLA PKA inhibition 6 h following reactivation has no effect on subsequent cue-and drug-induced reinstatement

All cannulae were implanted 1 mm above the BLA. Infusion numbers of acquisition sessions of heroin self-administration were not different between the groups that followed infusion with Rp-cAMPS (*N* = 8) or vehicle (*N* = 8) after reactivation [main effect of the trial session: *F*_(9,126)_ = 23.87, *p* < 0.001; main effect of the treatment condition: *F*_(1,14)_ = 0.003, *p* = 0.956; interaction of trial session × treatment condition: *F*_(9,126)_ = 0.583, *p* = 0.809; Figure 4B]. There are no differences between the groups across the 11 days of extinction session [main effect of the trial session: *F*_(10,140)_ = 77.31, *p* < 0.001; main effect of the treatment condition: *F*_(1,14)_ = 0.061, *p* = 0.808; interaction of trial session × treatment condition: *F*_(10,140)_ = 0.526, *p* = 0.870; Figure 4C]. There was no significant difference in the interaction between active nosepoke in the Rp-cAMPS and vehicle groups in the subsequent cue- (*p* > 0; Figure 4D) and drug-induced reinstatement test (*p* > 0.1; Figure 4E). This accords well with reconsolidation theory, the effect of Rp-cAMPS must occur within 6 h after the reactivation session.

## Discussion

The current study first, at least to our knowledge, confirmed the vital effect of PKA on the reconsolidation of heroin-associated memory. We found that intra-BLA infusion of Rp-cAMPS immediately after the reactivation of heroin-associated memory decreased cue- and drug-induced seeking behavior. Furthermore, intra-BLA infusion of Rp-cAMPS but not reactivation manipulation or infusion with a 6-h delay post-reactivation blocked the disruption of the reconsolidation of heroin-associated memory by Rp-cAMPS, certifying a key time window after reactivation during which the intra-BLA PKA inhibitor can interfere with the reconsolidation of the heroin-associated memory. In summary, we found that the disrupting reconsolidation of heroin-associated memory by a PKA inhibitor in BLA reduces subsequent heroin-seeking behavior, which is reactivation-dependent and time-limited. Moreover, such inhibition effect of heroin relapse lasted at least 28 days in the rats.

In the present study, we examined the role of PKA in the reconsolidation of heroin-associated memory *via* a classic heroin SA model. Intra-BLA infusion of a PKA inhibitor during heroin-associated memory reconsolidation (i.e., immediately after reactivation of memory) decreased cue-induced reinstatement of heroin-seeking behavior and lasted at least 4 weeks, relative to vehicle rats (Figures 1, 2). These observations indicate that PKA in BLA is necessary for heroin-associated memory reconsolidation in heroin self-administration, thus extending the knowledge of PKA in Pavlovian methamphetamine, morphine, cocaine, and amphetamine reward memories (Sharifzadeh et al., 2006; Gerdjikov et al., 2007; Arguello et al., 2014; Yang et al., 2020). Furthermore, our data in experiments 3 and 4, which show no effect of PKA inhibition on subsequent cue-induced heroin-seeking behavior when infusion is delayed time or without reactivation, support the reconsolidation theory, which states that the reconsolidation process is dependent on protein synthesis, which takes about 6 h after memory reactivation (Nader et al., 2000a,b; Quirk and Mueller, 2008). These results indicate that the effectiveness of PKA inhibition on the impairment of heroin-associated memory is retrieval-dependent and time-limited. The experiment results suggest that the activity of PKA in the BLA mediates the reconsolidation of heroin-associated memory, thus disrupting reconsolidation by Rp-cAMPS effectively reduces heroin-seeking behavior and preventing relapse in the rats.

Undeniably, we first found the potential therapeutic of PKA in heroin abuse disorders, but more studies should be done to reveal the underlying molecular mechanisms. The PKA holoenzyme of mammalians is composed of two regulatory and two catalytic subunits, that regulate learning and memory depending on the cAMP-responsive element binding protein (CREB) (Giese and Mizuno, 2013; Wang and Peng, 2016). The role of PKA signaling was first found in the formation of both short- and long-term memory in *Aplysia* (Dudai, 1988; Kandel, 2012). Furthermore, existing literature reported that PKA signaling can also affect the memory process of consolidation, reconsolidation, and extinction, as well as the behavioral counterpart of synaptic tagging (Goosens et al., 2000; Schafe and LeDoux, 2000; Isiegas et al., 2006; Young et al., 2006; Corcoran et al., 2013; Liu J. F. et al., 2015; Garcia-Pardo et al., 2016). In a recent study, Rabinovich Orlandi et al. found that infusing the PKA inhibitor during reconsolidation induced retrograde amnesia in the inhibitory avoidance and spatial version of the object recognition tasks, partly suggesting that the PKA activity is essentially required in the reconsolidation of different types of memory (Rabinovich Orlandi et al., 2020). The most relevant to the present study is that the bidirectional regulation of PKA during the reconsolidation in the BLA has the opposite role of cocaine cue memories, activation or inhibition of PKA in the reconsolidation process results in the enhancement or impairment of cocaine-seeking behavior, and the reinforcing properties of the CS, respectively (Sanchez et al., 2010; Arguello et al., 2014). But surprisingly, our data showed a significant difference in the heroin priming reinstatement test between the Rp-cAMPS and vehicle groups, which is inconsistent with Sanchez's et al. study with cocaine. In Sanchez's et al. study, the active lever numbers of PKA inhibition animals, to some degree but not statistical, increased in the cocaine-induced reinstatement test compared with vehicle animals. These different results may be because of the use of different drug classes (cocaine vs. heroin) and different procedures (duration of extinction days: 8 vs. 11 days; reinforcement behavior: lever press vs. nosepoke). In brief, the inhibition effects of a PKA inhibitor on memory reconsolidation across drug classes and paradigms are particularly exciting for the treatment of substance use disorders.

For the inconsistent results, the role of PKA in reconsolidation should be discussed in more detail. PKA is involved in the reconsolidation of aversive memory (Koh and Bernstein, 2003). Our data demonstrate the negative effect of PKA inhibition in the reconsolidation of heroin-associated memory which is similar to the role of PKA in the reconsolidation of auditory fear memory, suggesting a conserved memory process likely regulating both appetitive and aversive stimulus memories. There are two main potential underlying mechanisms for interpreting such phenomena. One interpretation is that PKA works *via* the β-adrenergic receptor, as both appetitive and aversive memory reconsolidation required G protein-coupled β-adrenergic receptor, which is the stimulation target in adenylyl cyclase and promotes the formation of the second messenger cAMP that activates PKA (Arnsten et al., 2005; Liu X. et al., 2015). Some studies supporting the hypothesis suggest that intra-BLA infusion of β-adrenergic receptor stimulation enhances, while β-adrenergic receptor antagonism disrupts the reconsolidation of both heroin-associated memory and fear memory (Debiec and Ledoux, 2004; Otis et al., 2013). In addition, the β-adrenergic receptor antagonist affects the reconsolidation of the drug-related memory process in both heroin-used animal and clinical studies (Zhao et al., 2011; Chen et al., 2021a). Another alternative interpretation is that PKA action in a CREB-depend way according that the PKA activity is important to induce CREB-mediated gene transcription after conditioning (Ahi et al., 2004; Sindreu et al., 2007). Reduced activations of CREB result in a disruption of reconsolidation of cocaine-conditioned place preference and an eight-arm maze task (Roullet and Sara, 1998; Miller and Marshall, 2005). Some studies imply that PKA may, *via* the extracellular-signal regulated kinase (ERK), activate CREB in the amygdala to be involved in the reconsolidation of contextual fear memory (Treisman, 1996; Tronson and Taylor, 2007; Mamiya et al., 2009; Tronson et al., 2012).

Our findings demonstrate that the PKA activity plays a key role in the reconsolidation of heroin-associated memory *via* the heroin SA paradigm. Intra-BLA infusion of Rp-cAMPS immediately after a memory reactivation session disrupts the reconsolidation of heroin-associated memory, reduces subsequent drug seeking, and prevents relapse, which is retrieval-dependent and time-limited and depends on BLA. This study extends earlier reports that the PKA activity affects the reconsolidation of drug-related memory, yet the effectiveness in treating clinical populations remains to be tested. Future studies should clarify the underlying specific target and examine whether systemic PKA antagonism also applies for better clinical applications.

## Data availability statement

The original contributions presented in the study are included in the article/supplementary material, further inquiries can be directed to the corresponding author/s.

## Ethics statement

The animal study was reviewed and approved by Xiangya Hospital Ethics Committee, Xiangya Hospital (Changsha, China).

## Author contributions

YZ and HaoxL carried out the main experiments and developed the manuscript. TH, ZZ, and QL reviewed and edited the manuscript. HaoyL proposed the central idea, reviewed and edited the manuscript, and acquired funding. All authors contributed to the article and approved the submitted version.

## Funding

This work was financially supported by the National Natural Science Foundation of China (Grant No. 82101247) and the Natural Science Foundation of Hunan Province, China (Grant No. 2021JJ40999).

## Conflict of interest

The authors declare that the research was conducted in the absence of any commercial or financial relationships that could be construed as a potential conflict of interest.

## Publisher's note

All claims expressed in this article are solely those of the authors and do not necessarily represent those of their affiliated organizations, or those of the publisher, the editors and the reviewers. Any product that may be evaluated in this article, or claim that may be made by its manufacturer, is not guaranteed or endorsed by the publisher.
